# Energetics and Oxidative Status: Seasonal Variation in Blood Oxidative Stress Metrics in Four Species of Small Birds from a Cold Winter Climate

**DOI:** 10.1093/iob/obaf024

**Published:** 2025-05-28

**Authors:** A G Jiménez, C J Marolf, O R Gulseth, S K Anandan, D L Swanson

**Affiliations:** Department of Biology, Colgate University, Hamilton, NY 13346, USA; Department of Biology, University of South Dakota, Vermillion, SD 57069, USA; Department of Biology, University of South Dakota, Vermillion, SD 57069, USA; Department of Biology, University of South Dakota, Vermillion, SD 57069, USA; Department of Biology, University of South Dakota, Vermillion, SD 57069, USA

## Abstract

Birds that overwinter in temperate regions must be physiologically flexible to face the demands of living in a thermally fluctuating environment. Much of the previous literature on this topic focuses on whole-animal metabolic rates and corresponding cellular and molecular mechanisms that enable these birds to withstand the demands of changing environmental conditions. Basal and maximal shivering metabolic rates, as well as daily energy expenditure, typically increase in winter for small birds overwintering in cold climates, which might increase the production of reactive oxygen species (ROS) within mitochondria as a natural byproduct of aerobic metabolism. In this study, we measured summer to winter differences in oxidative balance in four species of resident passerine birds. Blood samples were taken from field-collected American goldfinch (*Spinus tristis)*, black-capped chickadee (*Poecile atricapillus*), house finch (*Haemorhous mexicanus*), and house sparrow (*Passer domesticus*) during the summer and winter of 2023–2024 in South Dakota, USA. We determined plasma total antioxidant capacity and lipid oxidative damage, and red blood cell activities of three antioxidant enzymes: catalase (CAT), glutathione peroxidase (GPx), and superoxide dismutase (SOD). Lipid oxidative damage was significantly lower in winter for three of four species, and total antioxidant capacity for all species was significantly lower in winter compared with summer. Across all species, CAT activity was significantly higher in summer than in winter. In contrast, SOD activity was significantly higher in winter than in summer for all species. We also found species-level differences across the two seasons. These data suggest that the higher thermoregulatory costs in winter do not result in consistently elevated oxidative damage or antioxidant capacities relative to summer in small resident birds in cold climates, despite previously demonstrated winter increases in metabolic rates and energy expenditure. Such a result might occur as a function of either a reduction in dietary antioxidants and/or uncoupling of ROS production and metabolism in winter relative to summer or may be related to oxidative costs associated with reproduction.

## Introduction

Vertebrate animals that overwinter in temperate regions must be physiologically flexible to face the demands of living in an environment with seasonal temperature changes (Seebacher 2009; Swanson 2010). Ectothermic vertebrates respond to cold by temperature-induced reductions in metabolic rates, whereas endotherms active under cold conditions increase metabolic rates, so despite similar regulatory pathways for metabolism and thermogenesis (Seebacher 2009), endotherms may differ from ectotherms in metabolic responses to cold. In addition, many mammals wintering in cold climates tend to respond to winter cold by an energy conservation strategy, including periods of hibernation or dormancy but also reduced activity and use of favorable microclimates for non-hibernators. Most small birds, however, tend to increase energy use in cold winter climates, so undergo a winter metabolic upregulation not necessarily evident in other vertebrate groups (see Swanson et al. 2017 for review). For these reasons, birds make an excellent study system for testing questions related to seasonal energetics and oxidative stress (OS).

Examining the mechanisms underlying the ability of birds to endure varying climates has been the subject of numerous studies in recent years (Liknes et al. 2002; Swanson and Liknes 2006; Sgueo et al. 2012; Petit et al. 2013, 2014; Oboikovitz and Swanson 2022; Swanson et al. 2022; Le Pogam et al. 2024). Previous work has identified physiological mechanisms that enable birds to withstand seasonally changing environmental conditions, including an upregulation of basal (BMR) and maximal cold-induced (M_sum_) metabolic rates in winter (Swanson 2010). In addition, daily energy expenditure in small birds from cold winter climates tends to be higher in winter than in summer, as opposed to seasonal trends for larger birds or those from milder winter climates (reviewed in Swanson 2010). Reactive oxygen species (ROS) occur as a normal byproduct of aerobic metabolism within mitochondria (Brown et al. 2009; Skrip and McWilliams 2016). Increases in whole-animal metabolic rates associated with higher energetic workloads, such as prolonged flight, often result in increased ROS production and/or increased oxidative damage in birds (Beamonte-Barrientos and Verhulst 2013; Cooper-Mullin and McWilliams 2016; Dick and Guglielmo 2019; McWilliams et al. 2021). Accrual of oxidative damage through metabolic ROS production can particularly affect birds, due to their reliance on fats to support long-distance flights, especially during migration (Skrip and McWilliams 2016), and to fuel prolonged shivering in cold winter climates (Vaillancourt et al. 2005; Swanson 2010).

ROS production tends to be minimized in cases where non-shivering heat production occurs via the uncoupling of the electron transport chain. The concept of birds using uncoupling as a source of non-shivering thermogenesis is controversial, as birds lack brown fat and the uncoupling protein (UCP1) used by mammals for this process (Mezentseva et al. 2008). An uncoupling protein gene is present in birds (avUCP), but a definitive conclusion regarding the function of this gene has not yet been determined (Pani and Bal 2022). Nord et al. (2021) recently explored mitochondrial function in red blood cells of coal (*Perparus ater*), blue (*Cyanistes caeruleus*), and great (*Parus major*) tits during the winter and found that increased uncoupling of the electron transport chain occurred in the winter phenotype of both coal and great tits. This uncoupling produces heat (lowering metabolic efficiency) and bypasses the production of ROS, which could influence potential oxidative damage from ROS in winter-acclimatized birds. However, the activity of uncoupling proteins can also lead to unpredictable changes in the OS system. For example, it can lead to non-linear relationships between whole-animal metabolism and ROS production (Bury et al. 2018; Demine et al. 2019).

The regulation of potential cellular and molecular damage resulting from the production of ROS is accomplished by antioxidant defense and damage repair systems, which are interchangeably referred to as either free-radical balance or oxidative-balance systems (Cohen et al. 2008; Skrip and McWilliams 2016). The OS system involves multiple types of antioxidant enzymes inside the cell that counteract superoxide anions (a destructive and short-lived type of ROS) (Monaghan et al. 2009). These enzymes include superoxide dismutase (SOD), which turns the superoxide anion into hydrogen peroxide. Catalase (CAT) and glutathione peroxidase (GPx) then work together to break down hydrogen peroxide to water. In addition, low molecular weight, chain-breaking antioxidant compounds are also present within cells and are able to neutralize the harmful effects of ROS without continuing the chain of reactivity (Cohen et al. 2008; Skrip and McWilliams 2016). These antioxidants are either produced by the body or obtained through the diet; some examples are glutathione, vitamin E, and vitamin C. Together, these antioxidants work to facilitate chain-breaking that functions in the defense against ROS (Cohen et al. 2008; Skrip and McWilliams 2016). Lipids, particularly unsaturated lipids, are highly susceptible to accumulating oxidative damage, especially in the inner mitochondrial membrane, where the electron transport chain and its enzymatic complexes are located (Hulbert 2010). While this threat of damage is present in any animal, birds selectively incorporate particular unsaturated fatty acids into their membranes (Skrip and McWilliams 2016). This increases the risk of oxidative damage to the membranes of birds.

Here, we explore whether oxidative balance in blood differs seasonally in four species of passerine birds, including American goldfinch *Spinus tristis*, black-capped chickadee *Poecile atricapillus*, house finch *Haemorhous mexicanus*, and house sparrow *Passer domesticus*, all of which are year-round residents in the cold winter climate of South Dakota (Tallman et al. 2002) and show winter increases in basal and maximal thermogenic (i.e., summit, M_sum_) metabolic rates (Cooper and Swanson 1994; Liknes et al. 2002; Swanson and Liknes 2006; Oboikovitz and Swanson 2022). These study species were chosen to cover a range of body masses, roosting behaviors, and breeding schedules for small resident birds in the cold climate of South Dakota, because all these factors could presumably influence thermoregulatory costs, energetic workloads, and oxidative balance. Mean body mass ranged from 12–14 g for goldfinches and chickadees to 22 g for house finches and 28 g for house sparrows, and since smaller individuals have greater surface area to volume ratios (SA:V), thermoregulatory demands may increase for smaller species (Schmidt-Nielsen 1984). Daily energy expenditure, a measure of energetic workload, is typically higher in winter than in summer for small birds wintering in cold climates (Swanson 2010), but workload may vary throughout the breeding season and is often highest during the brood provisioning period (Williams 1996; Wiersma et al. 2004; te Marvelde et al. 2011; Senécal et al. 2021, but see Williams 2018), so differences in energetic workloads between and within seasons may also contribute to oxidative status in birds.

We measured total antioxidant capacity (TAC), lipid oxidative damage in plasma, and the activities of CAT, GPx, and SOD in red blood cells of each species and seasonal phenotype. Limited research on seasonal variation in oxidative balance in overwintering birds residing in cold winter climates exists (Jimenez et al. 2020a; Ramírez‑Otarola et al. 2023), complicating our understanding of the impact of increased thermogenic demands from cold temperatures on oxidative balance in birds. If energetic workload is positively related to ROS production (Yap et al. 2017; Gutiérrez et al. 2019; Zagkle 2022; but see Jimenez et al. 2020a), we hypothesized that a higher level of oxidative damage might occur in the winter due to the increased metabolic rates associated with thermogenesis at cold temperatures and the higher daily energy expenditure. We also hypothesized, however, that antioxidant levels might increase in the winter to mitigate the consequences of elevated energetic workload, resulting in the same amount or less oxidative damage than in the summer months. Alternatively, because reproductive activities may also affect OS in birds (Wiersma et al. 2004; Alonso-Álvarez et al. 2010; Metcalfe and Monaghan 2013; Pap et al. 2015; Merkling et al. 2017, but see Constantini et al. 2014), we expected seasonal oxidative status to differ among species based on their reproductive schedule, with less seasonal variation in species engaged in high-cost reproductive activities, such as feeding young. If this is the case, we hypothesize that goldfinches might show less seasonal variation in oxidative status than the other three species because capture dates were in the primary nesting season, whereas those for the other three species were likely in the post-breeding period. We included separate within-season analyses to further examine the potential relationship between energetic workload and OS. Specifically, within-season analyses tested whether oxidative status might be related to cumulative progression of the breeding or hot temperatures in summer or acute and/or long-term cold temperatures in winter.

## Materials and methods

### Blood sample collection

The samples for this study were collected at sites near Vermillion, Clay County, South Dakota (SD, USA; 43°47′ N, 96°55′ W) in both winter (December–February) and summer (July–August). Average temperatures in Vermillion, SD, USA, in January are −1°C (average daily high) and −10°C (average daily low; https://weatherspark.com/y/9040/Average-Weather-in-Vermillion-South-Dakota-United-States-Year-Round). Corresponding average daily high and low temperatures in July for Vermillion, SD, USA, are 30°C and 18°C, respectively. Samples were collected in summer and winter to represent samples from the warm-acclimatized and cold-acclimatized phenotypes, respectively. All procedures were approved by the Institutional Animal Care and Use Committee at the University of South Dakota (Protocol Nos. 08-05-21-24C and 10-12-23-26E) and carried out under a federal Master Banding Permit (22199).

To obtain blood samples, all birds were captured using mist nets at multiple locations near and within Vermillion, SD, USA. Specific capture dates during the summer breeding season for each species were from 18 July to 11 August for American goldfinch, 13 July to 15 August for black-capped chickadee, 22 July to 15 August for house finch, and 8 July to 17 August for house sparrow. These capture dates are during the nesting season for American goldfinch, during the late-breeding or post-breeding periods for black-capped chickadee and house sparrow, and during the post-breeding period for house finch (Tallman et al. 2002; Drilling et al. 2018). After capture, a small blood sample (<150 µL) was collected by pricking the brachial vein with a 26-gauge needle. Blood was collected in a heparinized micro-capillary tube, after which blood samples were transferred to a micro-centrifuge tube and immediately placed on ice in a cooler until transport back to the lab within 1–5 h. At the lab, the blood samples were placed in a refrigerated centrifuge and spun at 3000 *g* for 6 min to separate the plasma from the red blood cells. The plasma was drawn off using a micropipette. Both the red blood cells and plasma samples were then placed into a −80°C freezer until being shipped on dry ice to Colgate University for further analysis. Sample sizes from summer and winter for the four study species were: American goldfinch (summer *n* = 10, winter *n* = 13), black-capped chickadee (summer *n* = 8, winter *n* = 10), house finch (summer *n* = 10, winter *n* = 11), and house sparrow (summer *n* = 10, winter *n* = 16). We weighed birds at capture with an Ohaus Model LS200 balance, sexed birds by plumage characteristics at both seasons (goldfinches, house finches, and house sparrows) or by presence of brood patch or cloacal protuberance in the summer (chickadees); we did not sex chickadees in winter. We aged birds in summer by skull ossification or plumage characteristics, but did not sex hatch-year birds. Sample sizes of hatch-year birds in summer included six black-capped chickadees, one house finch, and one house sparrow. Values for OS metrics for hatch-year birds overlapped with those for adult birds for all species and OS metrics, so we pooled values for adult and hatch-year birds for analyses.

### Measuring OS in blood samples

The ability of plasma to neutralize hypochlorous acid was measured using the commercial kit for the OXY-Adsorbent test, which revealed TAC of the plasma (Diacron International, Grosseto, Italy). Oxidative lipid damage was measured as the presence of circulating hydroperoxides, including products of lipid oxidation, by performing the d-ROMs test on plasma (Diacron International). These tests concur with methods used in previous studies designed to measure the oxidative balance in birds (Skrip and McWilliams 2016; Jimenez et al. 2019).

Commercially available assay kits (Cayman Chemicals, Ann Harbor, MI) were used to measure CAT (cat. No. 707002) and GPx (cat. No. 703102) activities, as well as SOD concentrations (cat. No. 706002), which have been used in other studies to estimate antioxidant capacity in red blood cells (Jimenez et al. 2019). From the gathered samples, 4 µL of red blood cells were diluted with 396 µL of 20 mM HEPES, 1 mM EGTA, and 90 mM mannitol buffer solution. After dilution, samples were vortexed prior to each assay. We then followed the manufacturer's protocol to determine the activities of each of these enzymes. All enzyme assays were run on the same day as sample dilution. Furthermore, we quantified total protein in each diluted RBC sample using a protein determination kit (Cayman chemicals cat. No704002) to standardize across samples (Jimenez et al. 2019).

### Statistics

Data were analyzed in RStudio (version 2024.10.31) using R (version 4.4.2). To remove any effect of scale, all data were mean-standardized by dividing by the mean. We used *t*-tests for seasonal comparisons of body mass for each study species. For each measure of OS, we performed a generalized linear model (GLM) with the *stats* package (part of the base R package). The response variable was an individual measure of OS (e.g., lipid oxidative damage) and each full model had the following predictor variables: species, season, sex, time to bleed (i.e., the time elapsed between the bird hitting the mist net and the initiation of the blood sample), and body mass. Interactions with season were included in the models comparing summer and winter data (season * species, sex, and body mass). All response variables except GPx had a right-hand skew. Therefore, models for lipid OD, CAT, TAC, and SOD were fitted using a gamma distribution. Model diagnostics from the *DHARMa* package, including residual plots and Kolmogorov–Smirnov test, were used to assess the goodness of fit (Hartig 2024). The *emmeans* package was employed to generate estimated marginal means, with pairwise comparisons used when interactions were significant, to analyze species differences.

We included sex as a predictor variable because differences in reproductive activities between sexes could lead to differences in ROS production (Merkling et al. 2017). Sex could not be determined for most black-capped chickadees and in such cases was denoted as unknown. When unknown sex drove the significance of sex in any model, sex was removed, and the model was rerun. This prevented redundancies with black-capped chickadee data. Time to bleed may affect plasma metabolite and corticosterone levels (Guglielmo et al. 2002, 2005; Romero and Romero 2002), which could impact oxidative balance (Cohen et al. 2008), so we included time to bleed as a predictor variable. Finally, we included body mass as a predictor variable because of the impact of body size on metabolic rates and thermoregulatory demands (Schmidt-Neilsen 1984). We included interaction terms for variables that might seasonally differ in their impact on OS variables (species, sex, and body mass).

To determine which predictor variables and interactions were most important, we used the R package “MuMIn” to perform an automated model selection process with the dredge function (Barton 2024). We obtained the best-fit model with this approach but also considered models with ΔAICc scores < 2 as competitive. We determined the strength of each best-fit model by extracting pseudo-*R*^2^ values (Cox and Snell) using the function rsq.lr for GLMs in the “rsq” package (Zhang 2024). Predicted values for supplemental covariate plots were extracted using the “ggeffects” package (Lüdecke 2018).

For winter and summer data separately, we further analyzed each measure of OS among species to determine which variables might be significantly correlated with winter or summer OS. We used dredge() model selection, as with the combined-season models. Predictor variables for within-season analyses included species, body mass, time to bleed, and date. Summer models further included sex, age, and an interaction between species and date. The date variable was included to test whether progression of the season (i.e., different stages of the breeding season in summer or cumulative cold exposure in winter) could be related to oxidative status. Because juveniles may differ in metabolic rates from adults and may not have a fully developed OS system (López-Arrabé et al. 2015; Herborn et al. 2016), we included age as a predictor variable for summer samples when age (hatch-year vs. after hatch-year) was discernable.

In the individual season models, we also included prior temperature (average daily minimum [winter] or maximum [summer] temperatures [South Dakota Mesonet: https://climate.sdstate.edu/] over the 3-day period prior to blood samples from the Beresford, SD, USA, weather station, 34 km northeast of Vermillion) as a predictor variable to test whether recent temperature variation is related to the OS system. We chose 3-day periods both because intervals of cold and warm temperatures during the winter are expected to increase in future climate projects so multi-day periods of similar temperatures may become more common (IPCC 2021) and because birds in cold winter climates may begin to show metabolic adjustment within a few days of exposure to temperature changes (Swanson and Olmstead 1999; Dubois et al. 2016).

## Results

Mean (± SE) body masses for the four study species in summer and winter, respectively, were 12.9 ± 0.2 g (*n* = 10) and 13.8 ± 0.3 g (*n* = 13) for American goldfinch, 12.5 ± 0.4 g (*n* = 8) and 12.6 ± 0.3 g (*n* = 10) for black-capped chickadee, 21.8 ± 0.5 g (*n* = 10) and 22.1 ± 0.3 g (*n* = 11) for house finch, and 28.4 ± 0.4 g (*n* = 10) and 28.3 ± 0.4 g (*n* = 16) for house sparrow. Only American goldfinch showed a significant seasonal difference (*t*_21_ = 2.619, *P* = 0.016; *P *> 0.05 for remaining species) with winter birds being 7.3% heavier than summer birds.

Results of model selection for each measure of the OS system can be found in the supplemental materials. Metrics for top models for seasonal differences, summer, and winter, compared with the null model for each OS metric, are in Tables 1–3. Higher *R*^2^ values and lower AICc scores indicate that in all but one model, the best-fit model was stronger than the null, though the degree of strength in predictors varied by OS metric. Generally, GPx and SOD had the lowest *R*^2^ values, indicating the weakest model fit. Models for winter-only data had the lowest Δ AICc, indicating that the variables in our GLMs had limited predictive power. Nonetheless, our results highlight marked seasonal and species variation in OS system measures, in addition to some changes throughout the summer or winter season.

**Table 1.** Seasonal GLM model comparison metrics are included for the best-fit model for each measure of the OS system, including LPO (lipid peroxidation), total antioxidant capacity (TAC), catalase activity (CAT), glutathione peroxidase activity (GPx), and superoxide dismutase concentration (SOD)

**OS Metric**	**Top model predictors**	***R* ^2^**	**AICc**	**Null AICc**	**Δ AICc**	**Null deviance**	**Null df**
LPO	Season + Species + Season*Species	0.638	112.1	184.6	72.5	144.517	84
TAC	Season + Body mass + Season*Body mass	0.823	−139.7	1.7	141.4	5.093	84
CAT	Season + Species	0.939	−84.4	138.9	223.3	33.969	84
GPx	Season + Species + Season*Species	0.241	120.4	129.2	8.8	21.857	84
SOD	Season + Species	0.393	−88.0	−53.5	34.4	2.594	84

*Note*: Metrics include *R*^2^ values for the best-fit model (Cox and Snell), and comparisons for AICc scores, and deviance for the best-fit model and the null model.

**Table 2.** Summer GLM model comparison metrics are included for the best-fit model for each measure of the OS system

**OS Metric**	**Top model predictors**	***R* ^2^**	**AICc**	**Null AICc**	**Δ AICc**	**Null deviance**	**Null df**
LPO	Species + Date	0.756	80.0	125.0	45.0	78.002	37
TAC	Body mass + Date	0.507	−35.1	−14.3	20.9	0.908	37
CAT	Species	0.467	23.2	41.1	17.9	2.415	37
GPx	Species	0.482	40.2	57.1	16.9	9.000	37
SOD	Date + Species*Date	0.653	−59.2	−32.9	26.3	1.015	37

*Note*: OS metric names and table metrics are the same as in Table 1.

**Table 3.** Winter GLM model comparison metrics are included for the best-fit model for each measure of the OS system

**Model**	**Top model predictors**	***R* ^2^**	**AICc**	**Null AICc**	**Δ AICc**	**Null deviance**	**Null df**
LPO	Bleed time	0.063	−23.9	−22.8	1.1	13.100	46
TAC	Intercept only	0.023	NA	−152.8	NA	15.152	46
CAT	Species + Prior weather	0.863	−117.7	−42.1	75.6	4.278	46
GPx	Date	0.048	75.0	75.4	0.3	12.563	46
SOD	Species + Date	0.252	−37.8	−32.2	5.6	1.149	46

*Note*: OS metric names and table metrics are the same as in Table 1.

### Combined-seasons models—seasonal differences

**Fig. 1.**
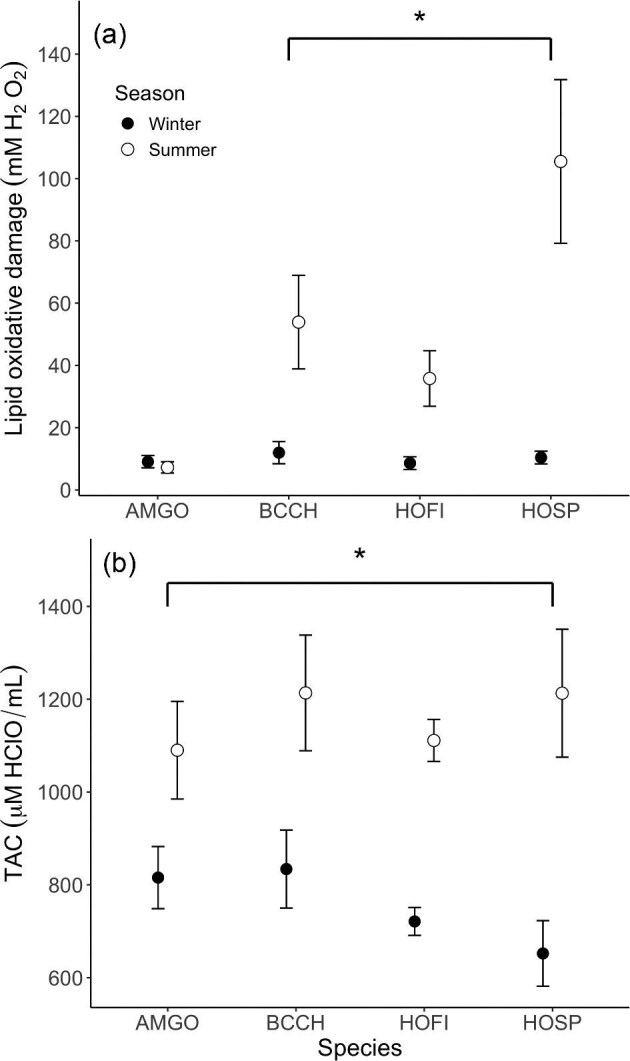
(a) Lipid oxidative damage for all species were significantly lower in winter compared with summer, and there were no differences across species in either summer or winter. (b) Total antioxidant capacity for all species was significantly lower in winter compared with summer. Total antioxidant capacity was significantly higher in house sparrows (HOSP) in winter compared with the three other species. Circles represent means, and error bars represent standard error around the mean (S.E.M.) Asterisks demonstrate differences between seasons.

The best-fit model for lipid oxidative damage retained main effects of season and species, and a season X species interaction term in the model (Table S1). When including the interaction term, our model estimates suggest that black-capped chickadee, house finch, and house sparrow all had greater lipid oxidative damage in summer than in winter, but lipid oxidative damage did not vary seasonally for American goldfinch (Fig. 1, Table S4). The best-fit model for total antioxidant capacity included main effects of season (greater in summer than in winter), body mass, and a season X body mass interaction term (Table S1). Correcting for the season X body mass interaction suggested that larger body mass is associated with higher TAC in summer, but not in winter. Though species was not a significant predictor in our top model, seasonal difference was greatest in house sparrows, which had summer levels of total antioxidant capacity higher than in other species (Fig. 1).

**Fig. 2.**
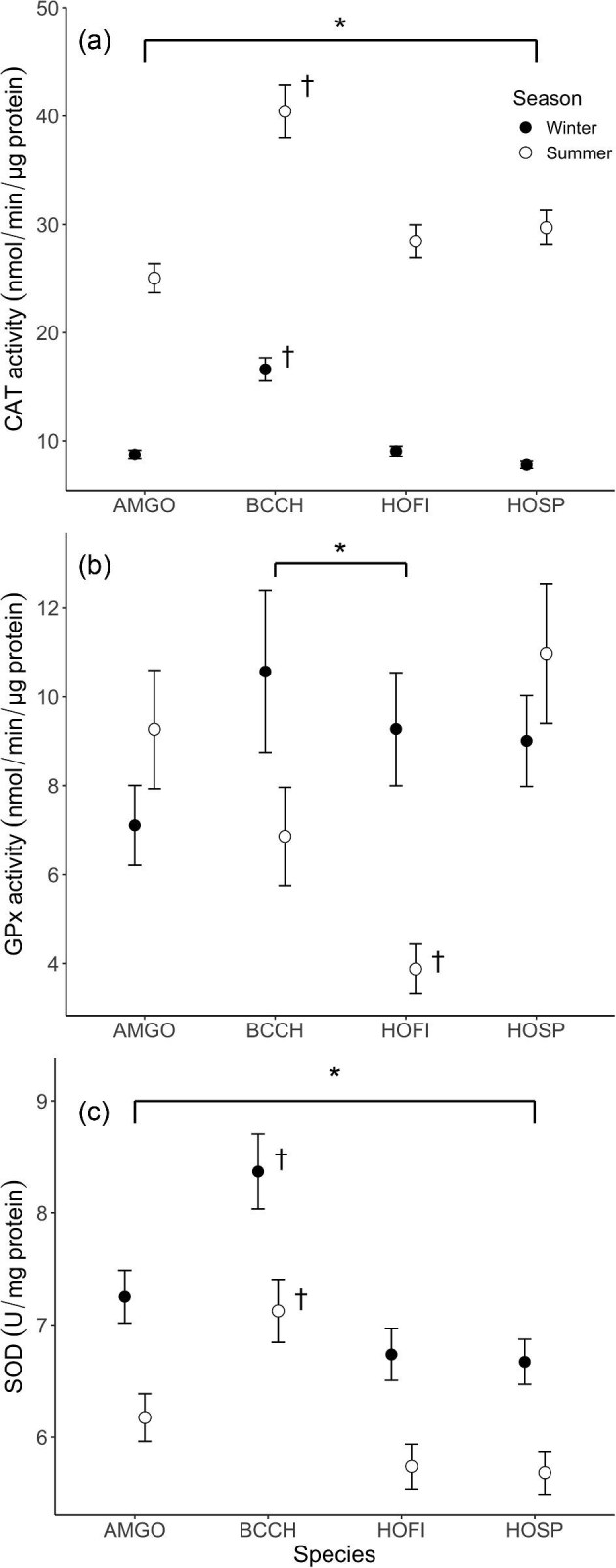
(a) CAT activity was significantly higher in the summer compared with winter. Additionally, black-capped chickadee (BCCH) CAT activity was significantly higher than the other three species in the summer and winter (b) GPx activity was significantly different across seasons for BCCH and house finch (HOFI). HOFI GPx activity was significantly lower than the other three species in the summer. (c) SOD concentration was significantly higher in the winter compared with summer and SOD concentration was significantly higher in BCCH compared with the three other species. Circles represent means, and error bars represent S.E.M. Asterisks demonstrate differences between seasons, while daggers (†) represent significant species differences.

The best-fit model for CAT activity included main effects for season and species with a species X season interaction term (Table S1). Some competitive models also included bleed time, sex, and body mass main effects, and a season X body mass interaction term (Table S1). CAT activity was significantly higher for all species in summer than in winter (Table S4), and black-capped chickadee showed higher CAT activity than the other three species in both seasons (Fig. 2). The best-fit model for GPx activity retained season, species, and a species X season interaction term in the model (Table S1). Across species, GPx activity was higher in winter than in summer for black-capped chickadee and house finch but was higher in summer than in winter, though not significantly, for American goldfinch and house sparrow (Fig. 2, Table S4). The only among-species difference occurred in summer, where house sparrows had higher GPx activity than other species (Fig. 2, Table S4).

The best-fit model for SOD concentration included main terms for season and species (Table S1). The other competitive model also retained body mass and a season X body mass interaction term in the model. In contrast to CAT activity, SOD concentration was significantly higher in winter than in summer for all species and was higher in black-capped chickadees than in other species at both seasons (Fig. 2).

### Individual-season models—prior temperature and date

#### Summer

The model containing date and species provided the best fit for summer lipid oxidative damage (Table S2). Some competitive models also included prior maximum temperature, age, and body mass main effects. In these models, lipid oxidative damage tended to decrease with date (Fig. S3). For total antioxidant capacity, the best-fit model retained main effects for date (higher TAC earlier in the summer, Fig. S4) and body mass (Table S2), with bleed time in a competitive model. Neither species (Fig. 1) nor prior temperature was included in any of the competitive models for TAC in summer.

The best-fit model for CAT activity in summer retained a main effect for species (Table S2). Black-capped chickadee CAT activity was significantly higher than in the other three species in summer (Fig. 2). Prior 3-day maximum temperature was included in some competitive models; an increase in temperature generally was generally associated with lower CAT activity. For summer GPx activity, the best-fit model included a main effect for species and competitive models also included effects of prior temperature, age, body mass, or date (Table S2). House sparrow GPx activity was significantly higher than in the other three species in the summer (Fig. 2). GPx activity tended to decrease as the summer season progressed and with higher 3-day maximum temperatures.

SOD concentration was higher in black-capped chickadees than in other species during the summer season (Fig. 2). The best-fit model for summer SOD concentration retained a main effect of date and species as well as the date X species interaction (Table S2). The negative effect of date was driven by American goldfinch (Fig. S5); the remaining species had a positive relationship or no relationship between progression of summer and SOD concentration. Summer SOD concentration was not strongly predicted by prior maximum temperatures.

#### Winter

The best-fit model for lipid oxidative damage in winter included only a main effect for bleed time, although the intercept-only model was competitive (Table S3). Neither prior temperature nor date was included in competitive models. There were also no significant among-species differences for lipid oxidative damage in winter (Fig. 1). For total antioxidant capacity, the best-fit model was the intercept-only model (Table S3). Date, prior temperature, and species (Fig. 1) were not included in any of the competitive models, so were not important predictors of TAC in winter.

The best-fit model for winter CAT activity included effects of species and prior minimum temperature (Table S3). Some competitive models also included positive effects of bleed time and/or date (Table S3). CAT activity tended to increase as the winter progressed. Winter black-capped chickadees demonstrated significantly higher CAT activity than the other three species (Fig. 2). CAT activity was generally positively correlated with higher minimum temperatures in winter (Fig. S7). The best-fit model for winter GPx activity included only a main effect of date, though the intercept-only model was competitive (Table S3), with generally higher GPx activity later in the winter season. The best-fit model for SOD concentration included species and date, and a competitive model also included body mass (Table S3). Winter SOD concentration was higher in black-capped chickadees relative to the other three species (Fig. 2). SOD concentration tended to increase with the progression of the winter season (Fig. S9).

## Discussion

Whole-animal metabolism and cell-level processes are linked across levels of organization through mitochondrial function, exemplified by the OS system, which balances mitochondrial ROS production and the potential damage accrued by this process (Hood et al. 2018; Havird et al. 2019). Our current study links previously measured differences in BMR, M_sum_, and daily energy expenditure in wild birds acclimatized to summer and winter phenotypes and explores underlying seasonal differences in the OS system.

### Seasonal differences

All four species included in this study, American goldfinch, black-capped chickadee, house finch, and house sparrow, are year-round residents in South Dakota (Tallman et al. 2002) and show winter increases in basal and maximal thermogenic (i.e., summit) metabolic rates (Cooper and Swanson 1994; Liknes et al. 2002; Swanson and Liknes 2006; Swanson et al. 2009; Oboikovitz and Swanson 2022). Moreover, small birds wintering in cold climates show increased daily energy expenditure in winter relative to summer (see Swanson 2010 for review). However, our data suggest an overall reduction in OS in winter months, contrary to the hypothesis that increased metabolic rates and energy expenditure would result in increases in the OS system. Most OS variables (except for SOD concentration) either decreased in winter compared with summer or were seasonally stable (GPx). Specifically, lipid oxidative damage and total antioxidant capacity for most or all species were significantly lower in winter compared with summer months, similar to prior results in multiple tissues from black-capped chickadees collected near Rimouski, Quebec, Canada (Jimenez et al. 2020a). Additionally, CAT activity in blood cells was significantly lower in winter compared with summer in all species. This implies that higher thermogenic costs, associated with elevated daily energy expenditure in the winter, are not directly linked to changes in these components of the OS system, a finding which further supports the notion that there is not a linear relationship between whole-animal metabolism and the OS system, as others have suggested (Bury et al. 2018; Demine et al. 2019).

Several possible explanations may account for the absence of a positive relationship between seasonal workload (i.e., daily energy expenditure) and oxidative status in our study, none of which are mutually exclusive. Firstly, the higher lipid oxidative damage and the increased variation in lipid oxidative damage in summer birds may reflect varying degrees of stress experienced during different portions of the breeding season, such as egg production, incubation, provisioning young, and defending territories. These different activities might be expected to result in varying workloads, especially among females, which might generate variation in OS dynamics (Lin et al. 2022).

Secondly, during the winter months in South Dakota, over-wintering birds have reduced access to dietary sources of antioxidants, such as ripe fruits, to buffer their endogenous TAC; thus, the observed winter decrease in circulating antioxidants could be partly due to reduced environmental availability. However, decreased TAC in the winter is not consistently supported in other studies. Ramírez-Otarola et al. (2023) found that two species of birds residing in a seasonal Mediterranean climate had increased TAC during the winter. In contrast, Sonmez et al. (2023) found that TAC was seasonally highest in spring (colder) in free-living common blackbirds (*Turdus merula*) residing in Turkey. Moreover, fruit consumption and increases in circulating antioxidant levels were not linked in various species of tropical birds (Jimenez and Cooper-Mullin 2022), and there is evidence that dietary shifts do not impact antioxidant function in free-living blackbirds (Ramírez‑Otarola et al. 2023). Thus, our study supports the idea that the availability of dietary antioxidants is not consistently related to oxidative status in birds.

Another potential explanation is that mitochondrial uncoupling may increase in winter birds along with increasing thermogenic requirements. When oxygen consumption and ATP generation are uncoupled, ROS production is reduced (Monaghan et al. 2009). The decreased TAC and lipid oxidative damage in winter-acclimatized birds in the present study may also be associated with uncoupling of mitochondria under conditions of elevated thermogenic demands. Expression of uncoupling proteins avUCP and adenine nucleotide translocase (ANT) increase in pectoralis muscle with cold acclimation in some birds (Pani and Bal 2022), although seasonal changes in gene expression for avUCP and ANT in pectoralis muscle do not occur in American goldfinches and black-capped chickadees (Cheviron and Swanson 2017). Non-shivering thermogenesis via ATP uncoupling, however, was associated with an increase in the mitochondrial volume of red blood cells during winter for three bird species in Sweden (Nord et al. 2021). This latter finding seems particularly relevant to our study since we also examined seasonal variation in the OS system in blood.

Another possibility is that despite ROS being a by-product of aerobic metabolism, daily energy expenditure and ROS production may not always be positively associated. Zhang and Wang (2021) argue that mitochondria are not the primary contributor to cellular ROS production under normal cellular conditions. Indeed, mitochondria-specific antioxidants may function to balance ATP production with ROS production so that a stable level of hydrogen peroxide is maintained across a wide range of aerobic workloads (Munro and Treberg 2017). If this is the case, the increased energetic demand for thermoregulation and the higher daily energy expenditure in winter relative to summer for small birds wintering in cold climates might not be expected to result in changes in oxidative status. Factors other than energetic workload that are associated with other life-history stages might result in the seasonal patterns in the OS system documented in this study.

Because fatty acid composition, specifically the degree of unsaturation, can influence susceptibility to oxidative damage (Pamplona et al. 1996, 1998, 2002; Portero-Otín et al. 2001; Hulbert et al. 2007), summer to winter variation in fatty acid composition of membranes could also contribute to the seasonal differences in oxidative status detected in this study. Seasonal and migratory variation in the relative proportions of saturated, monounsaturated (MUFA) and polyunsaturated (PUFA) in plasma, depot fat and other tissues occurs in birds, and these differences often reflect dietary differences among seasons (e.g., Andersson et al. 2015). Saturated fatty acids are sometimes higher in summer than in winter (Bower and Helms 1968; Carey et al. 1978; Andersson et al. 2015) but may be higher during winter than during migration (Egeler and Williams 2000; Klaiman et al. 2009), or seasonally stable (Zar 1977). Because PUFAs are particularly susceptible to oxidative damage, higher levels of PUFA in summer could explain the increases in oxidative damage and antioxidant capacity for summer birds in this study. Most evidence, however, suggests that PUFAs are seasonally stable (Zar 1977) or lower in summer than in winter (Bower and Helms 1968; Carey et al. 1978; Andersson et al. 2015), which is opposite of expectations if increased levels of PUFAs are driving the seasonal differences detected in this study.

Enzymatic antioxidants, CAT and SOD, demonstrated opposing patterns across seasons. CAT was higher in summer than in winter for all species, in agreement with muscle CAT activity in black-capped chickadees and rock pigeons living in central New York, which was highest in the summer months (Jimenez et al. 2020b). The opposite seasonal pattern occurred for SOD concentration, similar to results obtained for black-capped chickadees acclimatized to cold in Canada (Jimenez et al. 2020a). SOD catalyzes the conversion of oxygen free radicals to hydrogen peroxide, followed by conversion of hydrogen peroxide to water and oxygen by both CAT and GPx (Skrip and McWilliams 2016). The finding that SOD increases during winter, while CAT is downregulated and GPx shows no consistent seasonal changes, suggests that the initial step of converting ROS to hydrogen peroxide is critical for winter birds, whereas the conversion of hydrogen peroxide to water and oxygen is important for summer birds. Perhaps the redox signaling role, where hydrogen peroxide is involved in redox signaling modulating cellular metabolism, proliferation, growth, and survival (Veal and Day 2011; Marinho et al. 2014; Sies 2017; Di Marzo et al. 2018), is more important for winter birds where cellular metabolic rates are often increased relative to summer (Swanson 2010), resulting in a lower demand to convert hydrogen peroxide to water and oxygen.

Links between OS metrics and metabolic rates might also differ among tissues in birds (e.g., Jiménez et al. 2024). Consequently, thermogenically active tissues, such as skeletal muscle, heart, and liver might potentially show different seasonal patterns of variation than blood. Mitochondrial respiration in red blood cells, however, is more uncoupled in winter than in summer for passerines in seasonal environments (Nord et al. 2021), suggesting that cellular respiration and oxidative balance in red blood cells might also be expected to vary seasonally.

### Within-season effects of date and prior temperature

We were not able to track individual birds from which we acquired blood samples during summer to determine the precise stage of the breeding season for these birds, which could complicate interpretations of seasonal OS system phenotypes in the present study. We did, however, include date in the summer-season models to attempt to account, at least partially, for potential differences among different stages of the breeding season. In the present study, lipid oxidative damage tended to decrease as the summer season progressed, suggesting that lipid oxidative damage may be repaired later in the breeding season in some birds. We also detected a negative influence of summer date on TAC in the present study, suggesting that lower antioxidants later in the breeding season could contribute to the higher lipid oxidative damage in summer than in winter in the present study, although the lower TAC later in the summer did not correspond to higher lipid oxidative damage in late summer. This result, along with the low level of lipid oxidative damage in summer goldfinches, which were sampled in the middle of the breeding season, whereas other species were sampled either at the end of the breeding season or during the post-breeding period, suggests that breeding season activities do not consistently produce plasma lipid oxidative damage across all study species. Such inconsistency of accrual of oxidative damage across the breeding season in birds is generally consistent with many other studies (Williams 2018). Some studies, however, failed to detect effects of breeding activities on increasing OS in birds (Metcalfe and Monaghan 2013; Constantini et al. 2014), although others have found increased oxidative damage and/or increased antioxidant capacity as a function of breeding activities, especially when breeding workload is high (Wiersma et al. 2004; Alonso-Alvarez et al. 2010; Pap et al. 2015; Merkling et al. 2017).

Progression of the winter season did not impact lipid oxidative damage, so the accrual of oxidative damage over the winter season does not seem to be a consistent pattern in this study. This is further emphasized by the absence of an effect of 3-day minimum temperatures prior to sampling on lipid oxidative damage. This result is consistent with the findings of (Jimenez et al. 2020a,b) on black-capped chickadees. Similarly, prior 3-day maximum temperatures were not related to lipid oxidative damage in summer birds in this study, suggesting that hot temperatures, at least over the range experienced in this study, which ranged from 21.2 to 34.3°C, do not result in cumulative lipid oxidative damage. Acute or chronic heat stress may or may not induce oxidative damage in different tissues in birds, but this question has been studied mainly in poultry (Oladokun and Adewole 2022), so generalizations to wild birds remain uncertain.

TAC tended to decrease with progression of the summer season, despite the different timing of reproductive activities in goldfinches from the other three study species. Nevertheless, this suggests that breeding activities or changes in diet across the summer season may reduce the total antioxidant capacity in the plasma (Costantini 2016; Lin et al. 2022). TAC did not vary as the winter season progressed. These data are consistent with previous data and suggest that plasma TAC does not vary substantially across the winter season for small birds in cold climates generally (see Ramírez-Otarola et al. 2023). Prior temperature did not affect TAC at either season, suggesting that cold minimum temperatures in winter and hot maximum temperatures in summer are not important effectors of TAC in small birds.

High temperatures in summer might be expected to increase or decrease antioxidant enzyme activities (Jimenez et al. 2020a,b), so the tendency for increased CAT activities at higher temperatures in summer suggests that heat exposure might result in increases in enzymatic antioxidant capacity. However, 3-day prior maximum temperature was not a predictor of GPx activity or SOD concentration, so hot temperatures, at least at levels experienced by birds during this study, do not uniformly modify antioxidant enzyme capacities.

Prior exposure to cold temperatures in the winter demonstrated some species-specific trends for CAT activity, but not for other antioxidant enzymes. CAT activity tended to increase at higher winter 3-day minimum temperatures, suggesting that cold temperatures reduced CAT activity. This pattern is consistent with increased thermoregulatory demands at colder temperatures trading-off with antioxidant production capacity, but 3-day minimum temperatures were not predictors of other antioxidant enzymes, suggesting that the temperatures birds encountered during the present study do not consistently induce trade-offs in antioxidant enzymes.

### Species differences

We recognize that four species are insufficient to provide definitive tests of ecological mechanisms driving species differences, so we focus here on potential ecological differences among the species in the present study that could drive species differences in oxidative status markers. Our hope is to generate hypotheses for future research that can provide more definitive tests of potential ecological drivers of oxidative status in birds.

We detected several species differences among OS metrics in this study and several factors might relate to these differences. These factors include, but are not limited to, body size, nest type, timing of reproduction, and diet. Chickadees and goldfinches are smaller (11–14 g) than house finches and house sparrows (20–30 g), so the higher mass-specific metabolic rates associated with small body size, especially in winter, could influence OS. Higher CAT activity in chickadees is consistent with this expectation, but other OS variables were not, so body size does not appear to be a primary determinant of interspecific differences. The finches are open cup nesters, whereas chickadees and house sparrows are secondary cavity nesters. Ectoparasites may or may not occur in higher numbers in re-used nests of cavity-nesting birds (Rendell 1992; Mazgajski 2007; Lopez-Arrabe et al. 2012) and ectoparasites may reduce avian antioxidant defenses in adults or nestlings (Lopez-Arrabe et al. 2015; Wegmann et al. 2015), although not in all cases (Garrido-Bautista et al. 2021). Again, however, OS metrics were not clearly or uniformly related to nest type across species in the present study. Goldfinches differ from the other three study species in their timing of reproduction in South Dakota (Tallman et al. 2002), with the nesting season in goldfinches being later in the summer. Given that birds in the present study were all captured from mid-July to mid-August, this period is either post-nesting or at the end of the nesting season for study species other than goldfinches, which were in the middle of the nesting period. Summer lipid oxidative damage, however, was lower in goldfinches than in other species and antioxidants were generally similar between goldfinches and other species in summer, which is not consistent with this hypothesis. Finally, chickadees eat more animal matter than other species at both seasons (Badyaev et al. 2020; Foote et al. 2020; Lowther et al. 2020; McGraw and Middleton 2020) and diet may impact antioxidant capacities in birds (Cohen et al. 2008), but chickadees did not consistently differ from other study species across OS metrics in this study, so this expectation was not supported.

### Conclusion and perspectives for future studies

In the current study, we found that wild birds in the winter phenotype in South Dakota had lower lipid oxidative damage, lower TAC and lower CAT activity, but higher SOD concentration. We did not measure metabolic rates of birds for which we collected blood samples in this study, so we cannot directly test the relationship between metabolic energy expenditure and oxidative status. Nevertheless, because winter phenotype birds of these species demonstrate higher BMR and M_sum_ compared with the summer phenotype (Cooper and Swanson 1994; Liknes et al. 2002; Swanson and Liknes 2006), these data are consistent with the idea that whole-animal metabolism and the OS system are not directly and positively linked in birds. In addition, these data support other studies suggesting that breeding season activities in birds may generate OS (Wiersma et al. 2004; Alonso-Alvarez 2010; Pap et al. 2015; Merkling et al. 2017). We also found species-specific differences in antioxidant enzymes, as have others previously (Jimenez et al. 2019, 2020b), that might be related to different aspects of species life-history. Future studies documenting changes in oxidative status across different stages of the reproductive cycle in both female and male birds are needed to determine if workload differences among reproductive phases contribute to seasonal patterns of oxidative status in birds. In addition, studies performing concurrent measures of energetic workload, mitochondrial membrane potential, expression of uncoupling genes, and membrane fatty acid composition across seasons and across different stages of the reproductive cycle are needed to examine whether such uncoupling under conditions of increased energetic workload is associated with mediation of oxidative status in birds.

## Supplementary Material

obaf024_Supplemental_File

## Data Availability

Raw data can be accessed by emailing the authors.
