# Supplementary material for: Energetics and Oxidative Status: Seasonal Variation in Blood Oxidative Stress Metrics in Four Species of Small Birds from a Cold Winter Climate
Source: Integr Org Biol. 2025 May 28;7(1):obaf024. doi: 10.1093/iob/obaf024 (PMC12138340; doi:10.1093/iob/obaf024)
Supplement: obaf024_Supplemental_File [file obaf024_supplemental_file.docx]

**Supplementary Materials**

**Results**

*Combined-Seasons Models – Covariates*

Body mass was positively related to TAC in summer but not in winter (Fig. S1). Total antioxidant capacity tended to decline as time between capture and bleeding increased. Males tended to have higher CAT activities than females in summer, but not in winter. CAT activity tended to decrease as time between capture and bleeding increased and increased with body mass. SOD concentration tended to decrease as body mass increased, but this relationship was evident in summer but not winter (Fig. S2).

*Individual-Season Models - Covariates*

Summer

The only variable retained in every competitive model for lipid oxidative damage was date, which had a strong negative effect (Fig. S3). Body mass was retained in several competitive models for lipid oxidative damage (Table 2) and were generally positively related to lipid oxidative damage. Age (higher in HY birds) was retained in some competitive models but was not included in the best-fit model (Table 2). A body mass effect was included in the best-fit model for total antioxidant capacity, with higher TAC at greater body mass. One competitive model also included a main effect for bleed time (higher TAC with greater bleed times).

Competitive models for CAT activity in summer included bleed time, with lower CAT activity at longer bleed times. Some competitive models for summer CAT activity (Table 2) included age (lower CAT activity in juveniles). Age was also included as a covariate in one competitive models of summer GPx activity, with juveniles having higher GPx activity than adults. The top model for SOD retained only date and a species*date interaction.

Winter

The best-fit model for lipid oxidative damage in winter included a main effect for bleed time, with lower oxidative damage at longer bleed times (Fig. S6). For total antioxidant capacity, the best-fit model was the intercept-only model (Table S3), but competitive models retained main effects for body mass, with higher TAC for larger birds, and bleed time, with greater TAC at shorter bleed times.

Competitive models for winter CAT activity included bleed time (Table S3). CAT activity was negatively correlated with bleed time for the small species, especially black-capped chickadees, but not for the larger species when a species X bleed time interaction was included in the model Fig. S8).

**Discussion**

*Effects of covariates*

The relationship of TAC to M_b_ differed among seasons, with a positive relationship in summer but not in winter. Higher TAC in smaller individuals might be expected because of the relatively higher mass-specific metabolic rates in smaller individuals, but this was not observed in the present study. Thus, M_b_ does not appear to be a prominent effector of TAC, at least in the present study.

Enzymatic antioxidants showed variable trends with M_b_ in the present study. Body mass was positively associated with GPx activity in winter, but not in summer. In contrast, SOD concentration was positively correlated with M_b_ in winter, but not in summer. Catalase showed no relationship with body mass in either season. Such variable trends of blood enzymatic antioxidants with body mass are consistent with other studies (Berglund et al., 2014; Marasco et al. 2021), suggesting that body mass is not a consistent predictor of blood enzymatic antioxidant capacity in birds generally.

Males and females had similar levels of total antioxidant capacity than females, in opposition to data collected from male free-living blackbirds in Turkey, which demonstrated an increase in total antioxidant capacity from autumn to spring, whereas females did not show any seasonal differences in total antioxidant capacity (Sonmez et al., 2023). Others have noted that males had lower total antioxidant capacity than females in captive red-legged partridges (*Alectoris rufa*) (Alonso-Alvarez et al., 2010) and in wild white-crowned (*Pseudopipra pipra*) and red-headed (*Ceratopipra rubrocapilla*) manakins (Gomes et al., 2019). Sexual differences in antioxidant enzyme activities could be related to sexual differences in breeding activities (Lin et al., 2022; Costantini 2024). Enzymatic antioxidants in this study showed very little difference between sexes. CAT activity was higher in males than in females in summer but not winter, and no sex effects on GPx or SOD activity at either season. The lack of differences in enzymatic oxidants is consistent with metabolic rates in males and females, which do not tend to differ among sexes (e.g., Arens and Cooper 2005). Thus, sexual differences in breeding activities do not seem to be a prominent nor consistent effector of blood enzymatic antioxidant capacities in birds.

Age also had variable effects on oxidative status for summer birds in this study. Lipid oxidative damage was higher in hatch-year than in adult birds, but total antioxidant capacity did not different between adult and hatch-year birds. In contrast, old birds had higher TAC than young birds in red-legged partridges (Alonso-Alvarez et al., 2010), although all birds in that study were at least a year old. Antioxidant enzymes showed slight variable trends with age in the present study, with CAT activity lower, GPx activity higher, and SOD activity not different between hatch-year and adult birds. Blood GPx activity did not vary between juvenile and adult great tits (*Parus major*) (Norte et al., 2009). In addition, old birds had lower or similar blood enzymatic antioxidant activities than young birds for three species of passerines (Berglund et al., 2014). Thus, as for sex, age also appears to have variable and inconsistent effects on blood oxidative status in birds.

Bleed Time Effects

Additionally, our data serves to caution future studies regarding OS measurements in wild animals. Though other stress metrics, such as corticosterone concentrations, have been studied in detail with regards to animal handling times, and multiple handling and bleeding events (Huber et al., 2021), our paper is the first, of which we are aware of, to demonstrate differences in the OS system associated with the time elapsed between capture and bleeding. Bleed time was included as a predictor in both combined-season and within-season models for at least some OS system metrics. For example, lipid peroxidation tended to increase with bleed time, though only in winter. Total antioxidant capacity in plasma showed variable seasonal trends, increasing with bleed time in summer, but decreasing in winter. CAT activity tended to decrease with increasing bleed time in summer and decrease in winter, but other enzymatic antioxidants did not show similar effects. Thus, bleed time appears to induce confounding effects on at least some OS system metrics in wild birds. Therefore, we suggest statistically correcting for bleed time and season in OS datasets from wild birds.

**Supplemental Tables**

**Table S1**. Each line represents a competitive combined-season GLM models and model comparison values for the different OS system metrics in blood. Models are ranked by AIC score. OS metrics included LPO (lipid peroxidation), TAC (total antioxidant capacity), catalase activity (CAT), glutathione peroxidase activity (GPx), and superoxide dismutase concentration (SOD). Coefficients with multiple species interactions denote direction of slope with + or –, or +/– when the direction varied by species. The reference for season is summer, and therefore negative values reflect a winter decrease in the response variable. The reference species in all models was American goldfinch (AMGO).

| **OS Metric** | **Season** | **Species** | **Sex (M)** | **Body mass** | **Bleed time** | **Season**  ***Species** | **Season**  ***Sex** | **Season**  ***Body mass** | **df** | **AIC** | **Delta** |
| --- | --- | --- | --- | --- | --- | --- | --- | --- | --- | --- | --- |
| LPO | -1.258 | + | NA | NA | NA | – | NA | NA | 9 | 114.5 | 0.00 |
| TAC | -0.281 | NA | NA | 0.182 | NA | NA | NA | -0.160 | 5 | -138.9 | 0.00 |
|  | -0.283 | NA | NA | 0.198 | 0.017 | NA | NA | -0.166 | 6 | -138.7 | 0.20 |
| CAT | -1.052 | + | NA | NA | NA | +/– | NA | NA | 9 | -82.00 | 0.00 |
|  | -0.699 | + | NA | 0.271 | NA | NA | NA | -0.436 | 8 | -81.90 | 0.11 |
|  | -0.687 | + | + | 0.280 | NA | NA | NA | -0.453 | 10 | -81.10 | 0.91 |
|  | -1.051 | + | + | NA | NA | NA | NA | NA | 11 | -80.80 | 1.23 |
|  | -0.693 | + | NA | 0.214 | -0.019 | NA | NA | -0.430 | 9 | -80.40 | 1.58 |
|  | -0.955 | + | + | NA | NA | + | – | NA | 12 | -80.20 | 1.77 |
| GPx | -0.259 | +/– | NA | NA | NA | + | NA | NA | 9 | 122.8 | 0.00 |
| SOD | 0.161 | +/– | NA | NA | NA | NA | NA | NA | 6 | -86.9 | 0.00 |
|  | -0.019 | +/– | NA | 0.080 | NA | NA | NA | 0.177 | 8 | -86.5 | 0.39 |

**Table S2**. Best-fit summer GLM models and test statistics for the different OS system metrics in blood. OS metrics included LPO (lipid peroxidation), TAC (total antioxidant capacity), catalase activity (CAT), glutathione peroxidase activity (GPx), and superoxide dismutase concentration (SOD). Coefficients with multiple species interactions denote direction of slope with + or –, or +/– when the direction varied by species. The reference species in all models was American goldfinch (AMGO).

| **OS Metric** | **Species** | **Age (HY)** | **Body mass** | **Bleed time** | **Prior weather** | **Date** | **Species**  ***Date** | **df** | **AIC** | **Delta** |
| --- | --- | --- | --- | --- | --- | --- | --- | --- | --- | --- |
| LPO | + | NA | NA | NA | NA | -4.350 | NA | 6 | 82.2 | 0.00 |
|  | + | NA | NA | NA | 2.802 | -4.347 | NA | 7 | 82.3 | 0.05 |
|  | NA | 0.910 | + | NA | NA | -4.141 | NA | 5 | 82.3 | 0.07 |
|  | + | NA | NA | NA | NA | -1.087 | – | 9 | 82.8 | 0.62 |
|  | NA | 0.816 | + | NA | 2.056 | -4.109 | NA | 6 | 83.8 | 1.55 |
|  | + | 0.482 | NA | NA | NA | -4.261 | NA | 7 | 84.2 | 1.97 |
| TAC | NA | NA | 0.106 | NA | NA | -0.352 | NA | 4 | -35.0 | 0.00 |
|  | NA | NA | NA | NA | NA | -0.389 | NA | 3 | -33.8 | 1.13 |
|  | NA | NA | 0.134 | 0.024 | NA | -0.321 | NA | 5 | -33.3 | 1.71 |
| CAT | + | NA | NA | NA | NA | NA | NA | 5 | 25.1 | 0.00 |
|  | + | NA | NA | -0.078 | NA | NA | NA | 6 | 25.4 | 0.36 |
|  | + | -0.146 | NA | NA | NA | NA | NA | 6 | 25.9 | 0.81 |
|  | + | NA | NA | NA | -0.430 | NA | NA | 6 | 26.5 | 1.41 |
|  | + | NA | NA | -0.081 | -0.461 | NA | NA | 7 | 26.7 | 1.63 |
| GPx | +/– | NA | NA | NA | NA | NA | NA | 5 | 42.1 | 0.00 |
|  | +/– | NA | NA | NA | -0.821 | NA | NA | 6 | 43.5 | 1.45 |
|  | +/– | 0.218 | NA | NA | NA | NA | NA | 6 | 43.7 | 1.61 |
|  | +/– | NA | -0.239 | NA | NA | -0.239 | NA | 6 | 44.0 | 1.90 |
| SOD | NA | NA | NA | NA | NA | -0.381 | + | 9 | -52.7 | 0.00 |

**Table S3**. Best-fit winter GLM models and test statistics for the different OS system metrics in blood. OS metrics included LPO (lipid peroxidation), TAC (total antioxidant capacity), catalase activity (CAT), glutathione peroxidase activity (GPx), and superoxide dismutase concentration (SOD). Coefficients with multiple species interactions denote direction of slope with + or –, or +/– when the direction varied by species. The reference species in all models was American goldfinch (AMGO).

| **Variable** | **Species** | **Body mass** | **Date** | **Bleed time** | **Prior weather** | **df** | **AIC** | **Delta** |
| --- | --- | --- | --- | --- | --- | --- | --- | --- |
| LPO | NA | NA | NA | -0.125 | NA | 3 | -23.3 | 0.00 |
|  | NA | NA | NA | NA | NA | 2 | -22.5 | 0.79 |
| TAC | NA | NA | NA | NA | NA | 2 | -152.6 | 0.00 |
|  | NA | 0.022 | NA | NA | NA | 3 | -151.1 | 1.49 |
|  | NA | NA | NA | -0.005 | NA | 3 | -150.8 | 1.78 |
| CAT | +/– | NA | NA | NA | -0.078 | 6 | -110.4 | 0.00 |
|  | +/– | NA | 0.179 | 0.039 | -0.077 | 8 | -110.2 | 0.17 |
|  | +/– | NA | NA | 0.033 | -0.100 | 7 | -110.2 | 0.25 |
|  | +/– | NA | 0.218 | NA | NA | 6 | -109.6 | 0.85 |
|  | +/– | NA | 0.144 | NA | -0.057 | 7 | -109.5 | 0.89 |
| GPx | NA | NA | 0.393 | NA | NA | 3 | 75.6 | 0.00 |
|  | NA | NA | NA | NA | NA | 2 | 75.6 | 0.05 |
|  | NA | 0.147 | NA | NA | NA | 3 | 77.5 | 1.91 |
| SOD | +/– | NA | 0.148 | NA | NA | 6 | -35.7 | 0.00 |
|  | +/– | 0.405 | 0.124 | NA | NA | 7 | -34.3 | 1.45 |

**Table S4.** Estimated marginal means for estimates from seasonal GLMs in which season X species was a significant interaction. Metrics include lipid oxidative damage (Lipid OD), catalase activity (CAT) and glutathione peroxidase activity (GPx). Standard error (SE), degrees of freedom (df), and 95% confidence level intervals (CI) are reported alongside t-ratios which are all back-transformed from model log-transformations and are on the mean-standardized scale, such that estimates are compared to a null mean of 1.

| **Metric** | **Species** | **Season Contrast** | **Ratio** | **SE** | **df** | **Lower CI** | **Upper CI** | **t-ratio** | **p-value** |
| --- | --- | --- | --- | --- | --- | --- | --- | --- | --- |
| Lipid OD | AMGO | Summer/Winter | 0.796 | 0.264 | 77 | 0.412 | 1.54 | -0.688 | 0.4937 |
|  | BCCH | Summer/Winter | 4.502 | 1.84 | 77 | 1.999 | 10.14 | 3.689 | 0.0004 |
|  | HOFI | Summer/Winter | 4.16 | 1.43 | 77 | 2.096 | 8.26 | 4.14 | 0.0001 |
|  | HOSP | Summer/Winter | 10.134 | 3.22 | 77 | 5.384 | 19.07 | 7.291 | <.0001 |
| CAT | AMGO | Summer/Winter | 2.86 | 0.205 | 77 | 2.48 | 3.3 | 14.739 | <.0001 |
|  | BCCH | Summer/Winter | 2.43 | 0.214 | 77 | 2.04 | 2.9 | 10.128 | <.0001 |
|  | HOFI | Summer/Winter | 3.14 | 0.233 | 77 | 2.71 | 3.64 | 15.434 | <.0001 |
|  | HOSP | Summer/Winter | 3.82 | 0.261 | 77 | 3.33 | 4.38 | 19.587 | <.0001 |
| GPx | AMGO | Summer/Winter | 1.303 | 0.249 | 77 | 0.89 | 1.907 | 1.385 | 0.1702 |
|  | BCCH | Summer/Winter | 0.649 | 0.153 | 77 | 0.406 | 1.037 | -1.837 | 0.0701 |
|  | HOFI | Summer/Winter | 0.418 | 0.0831 | 77 | 0.282 | 0.621 | -4.387 | <.0001 |
|  | HOSP | Summer/Winter | 1.218 | 0.223 | 77 | 0.846 | 1.755 | 1.077 | 0.2849 |

**Supplemental Figures**


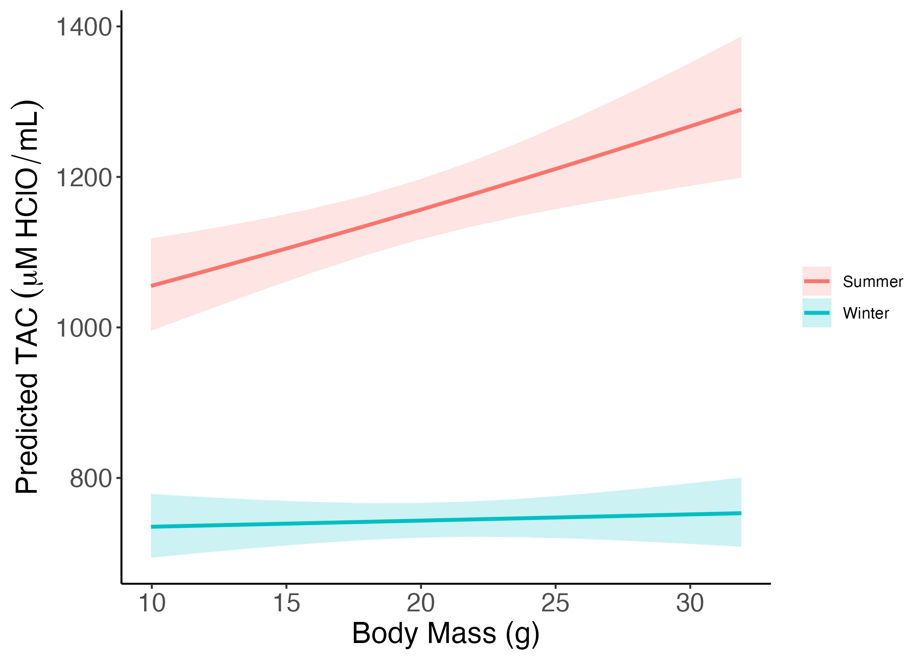


**Figure S1.** Total antioxidant capacity (TAC) varies with body mass between summer and winter for all species pooled. TAC activity tended to increase with body mass during summer but not winter. Trendlines show predicted values based on a GLM including season and body mass. Shaded areas represent 95% confidence intervals.


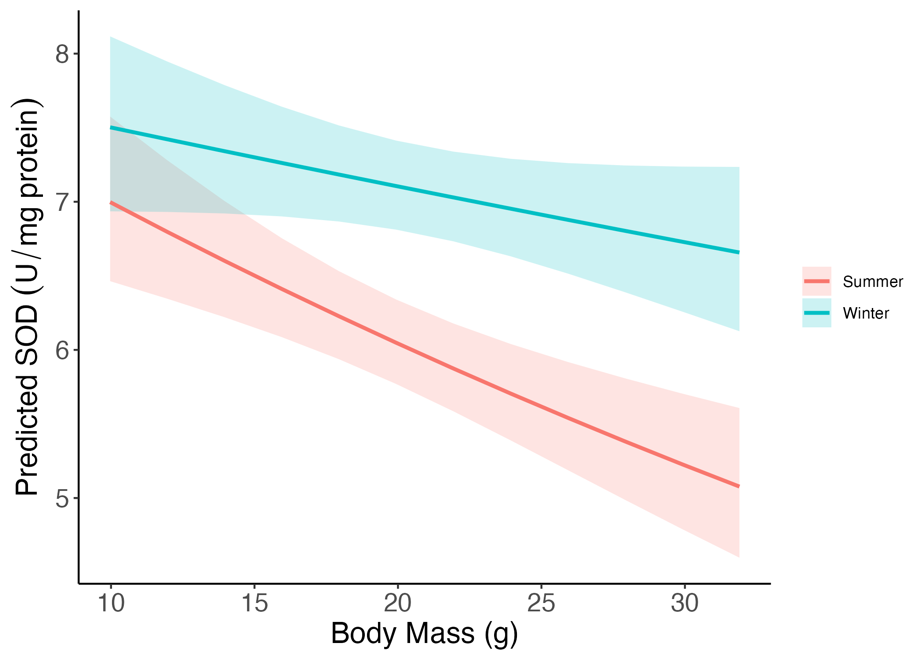


**Figure S2.** Superoxide dismutase (SOD) concentration varies with body mass in the summer for all species pooled. SOD concentration tended to increase with body mass during summer but not winter. This trend is not evident within species, and so the species effect was removed in this figure. Trendlines show predicted values based on a GLM including season and body mass. Shaded areas represent 95% confidence intervals.

**Supplemental Figures – Summer Models**


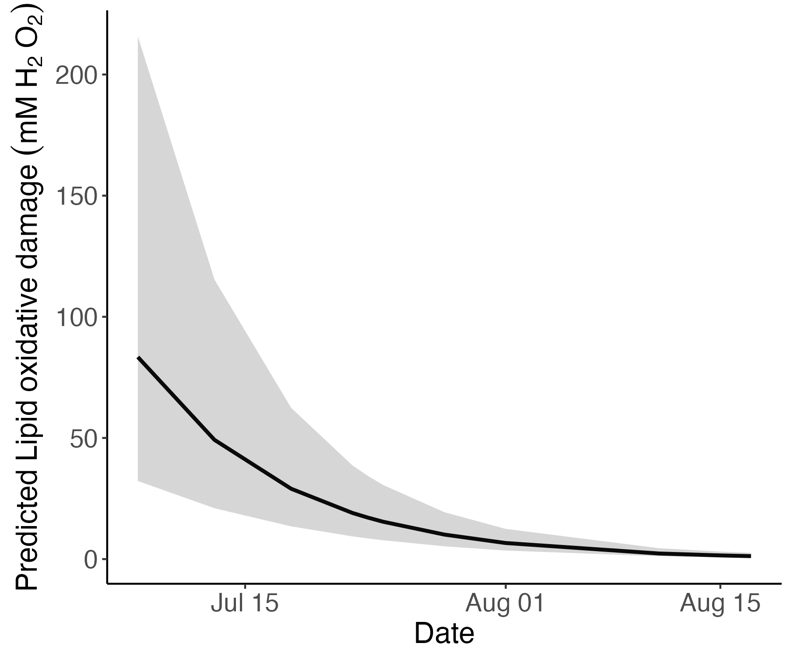


**Fig S3.** Lipid oxidative damage declines as the summer progresses when averaged across all species. The black line represents predicted lipid oxidative damage from a GLM including date and species as fixed effects. The shaded area represents the 95% confidence interval.

**
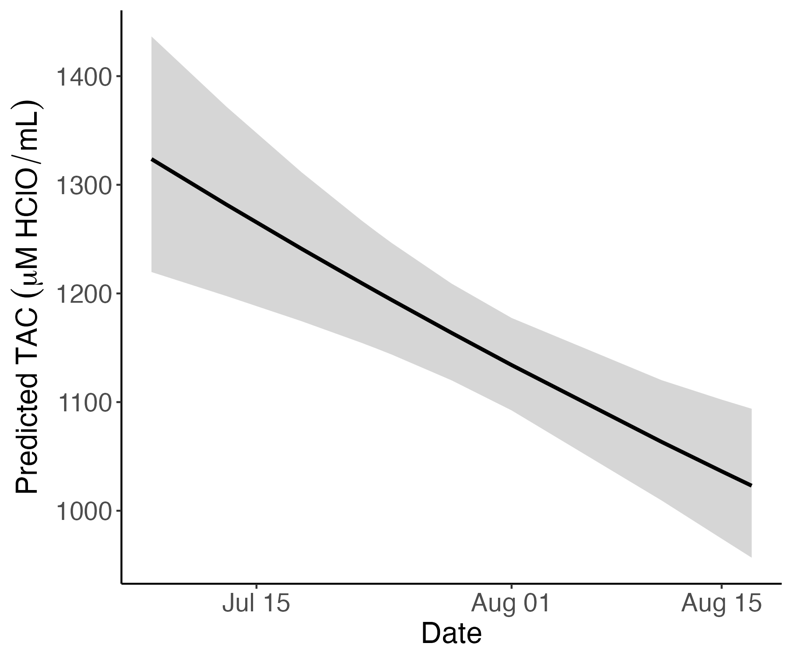
**

**Fig S4.** Total antioxidant capacity (TAC) declines as the summer progresses when averaged across all species. The black line represents predicted lipid oxidative damage from the top summer GLM for TAC, including date body mass, and bleed time as fixed effects. The shaded area represents the 95% confidence interval.


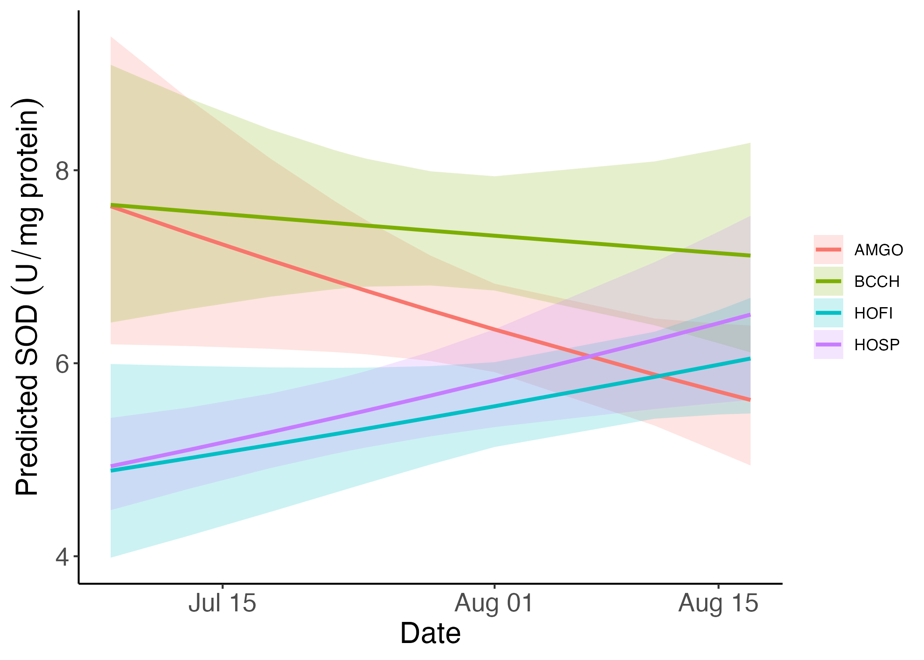


**Fig S5.** Superoxide dismutase (SOD) concentration varies by species over the summer. In smaller species, primarily American goldfinch (AMGO), SOD declines as the summer progresses. Larger species (HOFI and HOSP), tend to see an increase in SOD later in the summer. The solid lines represent predicted SOD from the top summer GLM for SOD. The shaded areas represent the 95% confidence interval.

**Supplemental Figures – Winter Models**


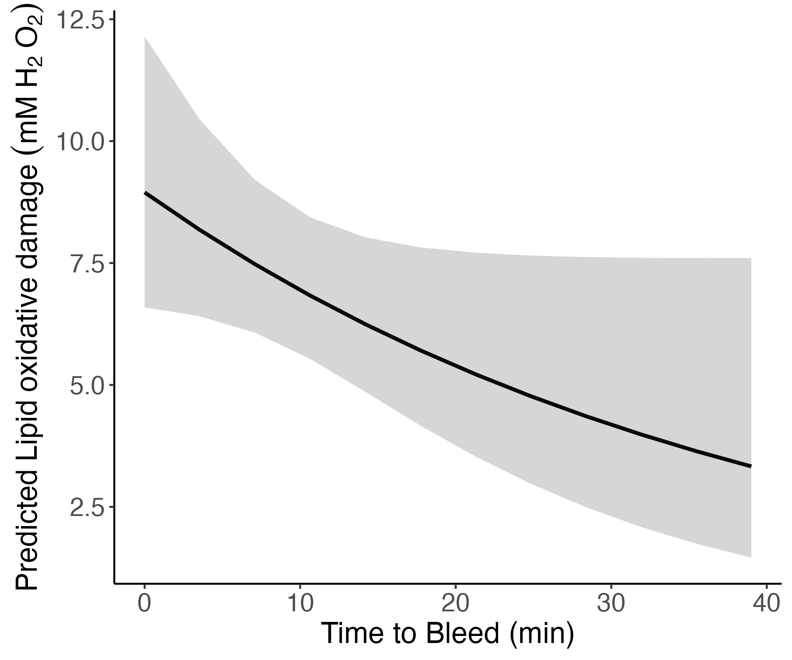


**Fig S6.** Lipid oxidative damage declines as bleed time increases when averaged across species. The black line represents predicted lipid oxidative damage from a GLM including date and species as fixed effects. The shaded area represents the 95% confidence interval.


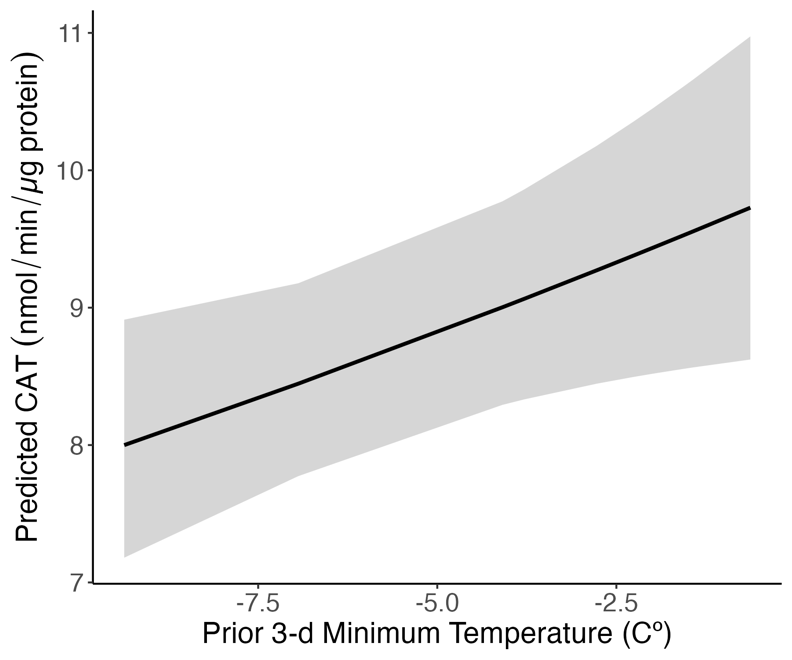


**Fig S7.** Catalase (CAT) activity declines as minimum temperatures decline in the winter across all species. The black line represents predicted lipid oxidative damage from the top summer GLM for CAT, including species as a fixed effect. The shaded area represents the 95% confidence interval.


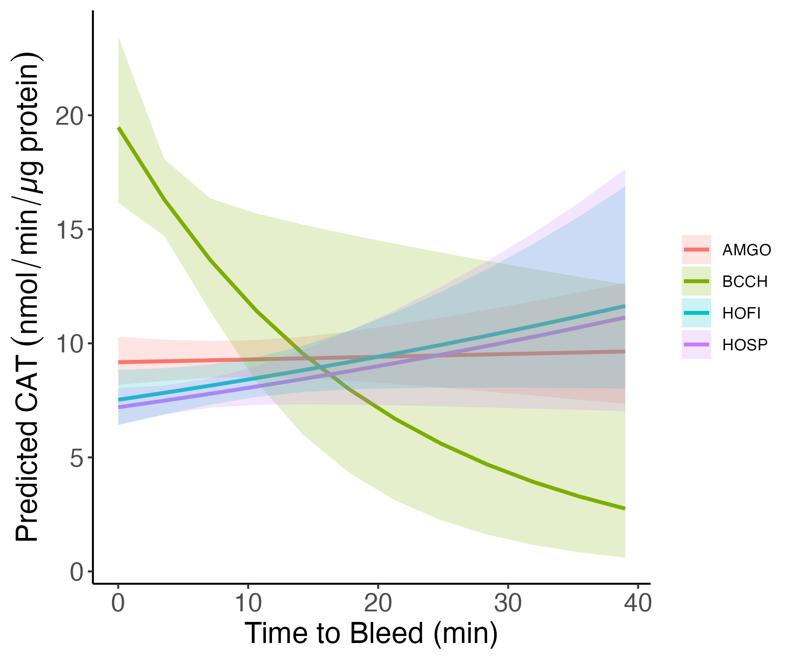


**Fig S8.** Catalase (CAT) activity declines as bleed time increases for black-capped chickadees (BCCH), but not for other species. The lines represent predicted CAT from a GLM including species, minimum 3-d prior temperature, and date as fixed effects. The shaded area represents the 95% confidence interval.


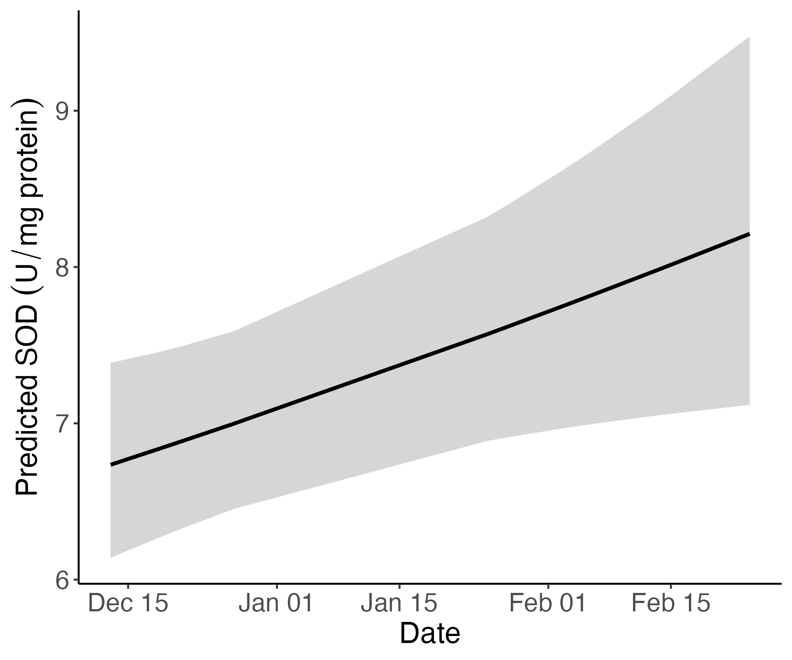


**Fig S9.** Superoxide dismutase (SOD) concentration increases as the winter progresses when averaged across all species. The black line represents predicted SOD from the top winter GLM for SOD, which includes date and species as fixed effects. The shaded area represents the 95% confidence interval.
